# P52 NHS Pharmacy First service: evaluating antimicrobial stewardship through WHO AWaRe classification and NICE guideline concordance in England (2024–2025)

**DOI:** 10.1093/jacamr/dlag102.058

**Published:** 2026-06-26

**Authors:** Sawsan Al Jundi, Rasha Abdelsalam Elshenawy

**Affiliations:** School of Health, Medicine and Life Sciences, University of Hertfordshire, Hatfield, UK; School of Health, Medicine and Life Sciences, University of Hertfordshire, Hatfield, UK

## Abstract

**Background:**

The NHS Pharmacy First service, launched in January 2024, represents a significant expansion of community pharmacy practice in England, enabling pharmacists to assess and supply antibiotics for seven defined clinical conditions under Patient Group Directions. Introduced by NHS England to reduce pressure on general practice, the service has important implications for antimicrobial stewardship given the volume of antibiotics supplied in primary care [1]. The UK Health Security Agency (UKHSA), the WHO AWaRe classification, and NICE Clinical Knowledge Summaries (CKS) provide key benchmarks against which prescribing quality can be evaluated [2–3].

**Objectives:**

To evaluate consultation activity across the seven NHS Pharmacy First clinical pathways; to assess antibiotic prescribing frequency and alignment with the WHO AWaRe classification; to examine concordance with NICE CKS first-line recommendations; and to describe demographic patterns of service utilization.

**Methods:**

A secondary data analysis was conducted using two publicly available national datasets covering February 2024 to November 2025. Consultation volumes were sourced from the NHSBSA Pharmacy First Clinical Pathways dataset. Antibiotic prescribing and demographic data were obtained from the OpenSAFELY Pharmacy First Monthly Dashboard, covering approximately 40% of the English population via TPP SystmOne practices. Antibiotics were classified using the WHO AWaRe framework and assessed against NICE CKS first-line recommendations. Ethical approval was not required, as all data were publicly available and anonymized.

**Results:**

Over the 22-month study period, 4 815 371 consultations were recorded across the seven clinical pathways. Acute sore throat was the most frequently accessed pathway (*n*=1 618 558; 33.6%), followed by urinary tract infection in women (*n*=1 293 763; 26.9%). A total of 232 055 antibiotic items were supplied. Prescribing was predominantly within the Access category (92.4%; *n*=214 485), substantially exceeding both the WHO 2030 target of greater than or equal to 70% and the England national benchmark of 64.1%. Phenoxymethylpenicillin was the most frequently supplied antibiotic (*n*=128 415; 55.3%), consistent with first-line recommendations for sore throat and sinusitis. Watch antibiotic use remained limited (7.6%), restricted to clarithromycin where clinically indicated, and no Reserve antibiotics were recorded. Prescribing was fully concordant with NICE CKS first-line recommendations across all six antibiotic-supplying pathways. Regarding demographics, female patients accounted for 67.3% of antibiotic-associated consultations, largely driven by the UTI pathway. Notably, communities with greater health equality needs demonstrated the highest engagement with the service (IMD 1: 26.9%) compared with the least (IMD 5: 14.9%), highlighting Pharmacy First's valuable role in improving equitable access to timely, guideline-driven care.

**Conclusions:**

NHS Pharmacy First has demonstrated itself as an effective model of community pharmacy-led care, achieving Access antibiotic prescribing of 92.4%, exceeding the WHO 2030 target and England national benchmark, with full concordance with NICE first-line recommendations across all pathways. These findings confirm that pharmacists can deliver safe, guideline-driven antibiotic supply at scale. Higher utilization among communities with greater health equality needs reflects the service’s potential to improve equitable access, supporting continued investment in Pharmacy First as an evidence-based model advancing the UK National Action Plan for AMR and reinforcing community pharmacists as frontline stewardship leaders.
